# Individual and interactive effects of drought and heat on leaf physiology of seedlings in an economically important crop

**DOI:** 10.1093/aobpla/plw090

**Published:** 2016-12-23

**Authors:** Honglang Duan, Jianping Wu, Guomin Huang, Shuangxi Zhou, Wenfei Liu, Yingchun Liao, Xue Yang, Zufei Xiao, Houbao Fan

**Affiliations:** 1 Jiangxi Provincial Key Laboratory for Restoration of Degraded Ecosystems & Watershed Ecohydrology, Nanchang Institute of Technology, Nanchang, Jiangxi, 330099, People’s Republic of China; 2 Department of Biological Sciences, Macquarie University, NSW 2109, Australia; 3 College of Water Conservancy and Ecological Engineering, Nanchang Institute of Technology, Nanchang, Jiangxi, 330099, People’s Republic of China

**Keywords:** heat stress, photosynthesis, recovery, stomatal conductance, water deficit, water relations

## Abstract

Heat waves in combination with drought are predicted to occur more frequently with climate warming, yet their interactive effects on crop carbon and water balance are still poorly understood. Hence, research on the capacity of crops to withstand and recover from the combined stress is urgently needed. This study investigated the effects of drought and heat wave on a crop species as well as the recovery from the combined stress. Seedlings were grown in growth chambers under two soil water conditions (*i.e*. well watered and drought stress) at ambient temperature (26 °C) for 10 days. Afterwards, half of the seedlings were exposed to a 7-day 42 °C heat wave. All the drought-stressed seedlings were then rehydrated upon relief of the heat wave. Leaf gas exchange, the maximum carboxylation capacity (*V*_cmax_), plant growth, relative chlorophyll content and leaf water potential were examined during the experimental period. The heat wave reduced leaf gas exchange rates, *V*_cmax_ and relative chlorophyll content, while it had no impacts on leaf water potential. In contrast, drought stress led to greater reductions in leaf gas exchange rates, growth and water potential than heat wave alone. Seedlings underwent a greater degree of stress in the combination of drought and heat wave than under the single drought treatment. The recovery of leaf gas exchange from drought stress lagged behind the water potential recovery and was delayed by heat wave. Our results show that drought stress had a predominant role in determining plant physiological responses and the negative impacts of drought stress were exacerbated by heat wave. The greater stress in the combination of drought and heat wave translated into the slower recovery of leaf gas exchange. Therefore, drought combined with heat wave may induce greater risks on crops under future climates.

## Teaser

This study investigated the combined effect of drought and heat wave on physiological responses of tomato seedlings. We found that the negative impacts of drought stress were exacerbated by heat wave through stomatal and biochemical limitations. Additionally, the recovery of leaf gas exchange in the combination of drought and heat wave was delayed more than in other treatments. Our results suggest that future climates characterized by drought and extreme heat may have substantial impacts on tomato productivity and survival.

## Introduction

Weather extremes, such as heat waves and severe droughts, are expected to increase in frequency and intensity under climate change (Reichstein *et al.* 2013; IPCC 2014). Plants appear to be more sensitive to weather extremes than to gradual changes in weather mean conditions, and therefore weather extremes may have more pronounced impacts on plants (Medvigy *et al.* 2010; De Boeck *et al.* 2011; Bauweraerts *et al.* 2013; Anderegg *et al.* 2015; Siebers *et al.* 2015). Despite the future co-occurrence of drought and heat waves, we still lack of evidence as to what degree the interactive effects of drought and extreme heat would affect the physiological responses of crops (De Boeck *et al.* 2011; Teskey *et al.* 2014). In particular, there is still limited information on how crops would recover from the combined stress of drought and heat wave. Tomato is recognized as the 4^th^ most valuable agricultural product worldwide (O’Carrigana *et al.* 2014). Hence, experimentally quantifying the tolerance of tomato to the combined extremes and the following recovery from stress can provide insights into better predicting tomato physiology and productivity in the future, thereby understanding how tomato would cope with climate change.

Plant carbon and water balance are both affected by drought and heat alone or in combination (Hartmann *et al.* 2013; Mitchell *et al.* 2013; Will *et al.* 2013; Zhao *et al.* 2013; Duan *et al.* 2014). On one hand, as the soil dries, plants close their stomata to reduce transpiration and water loss in order to avoid the failure of xylem water transport, while the consequent stomatal closure is accompanied with inhibition in growth and a reduction in carbon assimilation (McDowell *et al.* 2008; Woodruff *et al.* 2015). On the other hand, as air temperature and associated vapor pressure deficit (VPD) increase, plants can up-regulate transpiration for leaf cooling to prevent excessive heat damage (Ameye *et al.* 2012; Teskey *et al.* 2014). Nevertheless, larger evapotranspiration demands could induce soil water deficit and/or have negative feedbacks on stomatal conductance, thereby leading to stomatal closure and reduced photosynthesis (Duursma *et al.* 2014). In addition, once the thermal threshold is exceeded, non-stomatal limitations, such as degradation of chlorophyll, reduced activity of Rubisco activase and damage of photosystem II (PSII) could occur, further reducing carbon assimilation (*e.g*. Cunningham and Read 2006; Rennenberg *et al.* 2006; Haldimann *et al.* 2008; Hamilton III *et al.* 2008; Bauweraerts *et al.* 2014; Teskey *et al.* 2014; Wujeska-Klause *et al.* 2015).

Drought and extreme heat stress are usually thought to be highly interrelated. Each stress can exacerbate the stress severity of the other (Hamerlynck *et al.* 2000; Arnone *et al.* 2008; De Boeck *et al.* 2011; Ameye *et al.* 2012; Carmo-Silva *et al.* 2012; Bauweraerts *et al.* 2013; Teskey *et al.* 2014; Lobell *et al.* 2015; Ruehr *et al.* 2016). Drought stress is usually increased by heat (Zhao *et al.* 2013; Duan *et al.* 2014), while stomatal closure due to short term drought stress can prevent leaf cooling under heat and therefore push plants towards the thermal threshold (Teskey *et al.* 2014). Hence, the combined stress of drought and extreme heat would have more pronounced negative impacts on plants than the single factor. For instance, it has been demonstrated that photosynthesis in a herbaceous community was reduced to a greater extent in the combination of drought and heat wave than single drought or heat wave treatment (De Boeck *et al.* 2011). However, it has also been recognized that prolonged drought stress can improve the biochemical thermotolerance of plants (Chaves *et al.* 2002).

Unlike investigation on the resistance of plants to drought and heat wave, the recovery of plant physiological processes from the combination of the two stresses is far less studied. The recovery rate of leaf physiological responses may vary depending on the severity of previous stress and the legacy effects on plants (Anderegg *et al.* 2015; Mitchell *et al.* 2016). For example, one recent study has shown that recovery of photosynthesis in the combination of drought and heat stress was much slower than that in the single drought treatment even three weeks after the release of stress (Ruehr *et al.* 2016), mainly due to the irreversible photoinhibition under the combined stress. Additionally, information on the coordination of gas exchange and water relations during recovery from combined extreme stress remains scarce. Whether their relationships would be modified by the combined stress is still unknown. Consequently, more efforts are needed to investigate the capacity of plants to regulate carbon and water relations during the recovery following multiple stresses, providing more insights into implications for plant survival and productivity in the future real world.

This study aimed to examine the leaf physiology and growth of cherry tomato (*Solanum lycopersicum*) seedlings during the periods of combined stress of drought and heat wave and recovery following stress. Leaf gas exchange, plant growth, relative chlorophyll content, leaf water relations and the maximum carboxylation capacity (*V*_cmax_) were measured or estimated during the course of the experiment. The key research question is how plant carbon and water relations are coordinated during the recovery from the combined stress of drought and heat wave, which is crucial for tomato management strategies under climate change. We hypothesized that: (i) single heat wave treatment would lead to lower steady state leaf gas exchange rates, decreased water use efficiency, lower *V*_cmax_, reduced plant growth and more negative leaf water potential compared with ambient temperature control; (ii) heat wave would exacerbate drought stress by reducing steady state leaf gas exchange rates, *V*_cmax_, plant growth, leaf water potential, chlorophyll content to a greater extent in the combination of drought and heat wave treatment than in the single drought treatment; (iii) the coordination of leaf gas exchange and leaf water potential during recovery from drought would be shifted between ambient temperature and heat wave treatments, *i.e*. the recovery of leaf physiology from drought would be slower in the combination of drought and heat wave than in the single drought treatment.

## Methods

### Plant materials and growth conditions

Seeds of cherry tomato (*Solanum lycopersicum*; produced by Beijing Fangxuanyuan Seed Co, Ltd.) were sown and germinated in seed raising tube stocks in a poly-tunnel under ambient environmental conditions for one month. Healthy seedlings with similar heights (15–20 cm) were then transplanted into 3.7-L plastic pots filled with soil (one seedling per pot) and were grown for another month. Twenty four potted seedlings were then randomly assigned into two illuminated growth chambers (GS-1, Wuhan Ruihua Co, Ltd, China) (twelve seedlings per growth chamber) with a 26 °C/19 °C day/night temperature cycle and 15 h/9 h day/night photoperiod, 60% relative humidity (RH), and 250 µmol m^−^^2^ s^−^^1^ photosynthetic photon flux density (PPFD). The seedlings were well irrigated daily and fertilized weekly with a commercial liquid fertilizer (Soluble nutrient fertilizer, Demaisie Crop Science Co, Ltd, China). Seedlings were rotated within and between growth chambers every week to minimise potential effects of growth chambers on plant performance.

### Experimental design

**Figure 1:**
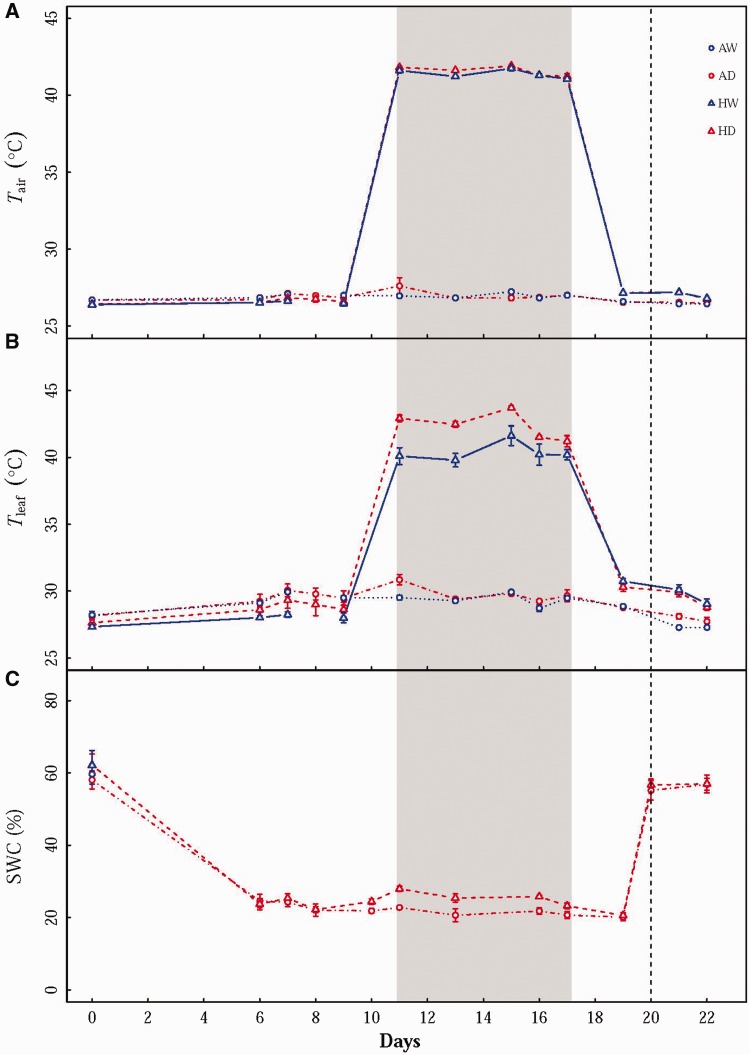
Measured (A) daytime air temperature (*T*_air_), (B) leaf temperature (*T*_leaf_) and (C) soil water content (SWC) at 8 cm depth throughout the experiment. Values are means ± SE (*n* = 4–6). The grey area represents the period during which a 7-day 42 °C heat wave was applied. The vertical line represents the day when droughted seedlings were rehydrated. Treatments: AW–Ambient temperature plus well watered; AD–Ambient temperature plus drought; HW–Heat wave plus well watered; HD–Heat wave plus drought.

Following 2 weeks of additional growth in the growth chambers, half of the seedlings (*i.e*. six seedlings) in each growth chamber were randomly assigned to two water treatments (*i.e*. well watered and drought treatments) on Day 0. The drought treatment consisted of one cycle of drought and recovery. Seedlings in the drought treatment in both growth chambers were treated under moderate drought stress until they were rehydrated on Day 20. During that time, the heat wave was imposed on one of the growth chambers for a period of 7 days. Specifically, from Day 10, one growth chamber was subjected to heat wave treatment (*i.e*. 42 °C/35 °C day/night temperature cycle), while the other one was maintained at ambient temperature treatment (*i.e*. 26 °C/19 °C day/night temperature cycle) (see Fig. 1A). The temperature in the heated growth chamber returned to ambient level on Day 17. Thus, this experiment was a factorial of 2 water × 2 temperature treatments, with six seedlings in each treatment. The four treatments in this experiment were: (1) Ambient temperature plus well watered (AW); (2) Ambient temperature plus drought (AD); (3) Heat wave plus well watered (HW); and (4) Heat wave plus drought (HD). Details of water and heat wave treatments are described below.

#### Water treatment

Seedlings in AW and HW treatments were maintained well watered throughout the experiment, while seedlings in AD and HD treatments were subjected to a target moderate drought stress and followed by rehydration. Given that measuring stomatal conductance (*g*_s_) is non-destructive, and that *g*_s_ is a good indicator of plant and leaf water stress, we controlled this variable in order to establish the target drought stress. Water was then withheld in seedlings in the drought treatment until *g*_s_ declined to the range of 0–0.05 mol m^−^^2^ s^−^^1^ (see Duan *et al.* 2014). By controlling *g*_s_ in this range, we established standardised drought stress across temperature treatments, ensuring that seedlings in AD and HD were subjected to similar drought stress throughout the experiment. When *g*_s_ followed into the range of 0–0.05 mol m^−^^2^ s^−^^1^, every pot in the drought treatment was weighed in the afternoon (between 1600 h and 1700 h) each day to determine gravitational water loss and the corresponding soil volumetric water content (SWC) (measured by a hand-set TDR with a depth of 8 cm; TZS-I, Zhejiang Topu Co. Ltd. China). About 50–100 ml water was then added to each pot to maintain the target SWC through the cycle of drought stress (25 % on average from Day 6 to Day 19) (see Fig. 1C). Note that the SWC in the well watered treatments was about 60 % on average. All seedlings in AD and HD were then rehydrated to well watered conditions on Day 20.

#### Heat wave treatment

On Days 10 and 11, the temperature in the heated growth chamber was raised from 26 °C to 42 °C in 4 steps, *i.e*. 30 °C, 34 °C, 38 °C, 42 °C and was maintained at each step for 4 h to allow plants to acclimate to the temperature changes. The temperature was then maintained at 42 °C/35 °C day/night before it declined back to 26 °C/19 °C day/night on Day 17. In this case, plants in HW and HD were subjected to a 7-day of + 16 °C heat stress treatment.

### Leaf gas exchange measurements

Leaf gas exchange measurements were taken on recently, fully expanded leaves from four seedlings per treatment (*n* = 4) using a portable open path gas exchange system (Licor-6400, Li-Cor, Lincoln, NE, USA) supplying photosynthetic photon flux density (PPFD) by red-blue light source (6400-02B). Leaf photosynthesis under saturating light (*A*_sat_, µmol m^−^^2^ s^−^^1^), stomatal conductance (*g*_s_, mol m^−^^2^ s^−^^1^), transpiration (*E*, mmol m^−^^2^ s^−^^1^), leaf-to-air VPD (VpdL, kPa), air temperature (*T*_air_) and leaf temperature (*T*_leaf_) were measured at mid-day (between 0930 h and 1400 h) throughout the experiment, at PPFD of 1200 µmol m^−^^2^ s^−^^1^, [CO_2_] of 400 µmol mol^−^^1^ and mid-day growth temperatures. Besides, the ratio of intercellular to atmospheric [CO_2_] (*C*_i_/*C*_a_) and the instantaneous water use efficiency (*WUE*_i_, μmol CO_2_·mol^−^^1^ H_2_O; determined by *A*sat*/g*s) were also calculated. Leaf dark respiration (*R*_d_, µmol m^−^^2^ s^−^^1^) was measured during the day after *A*_sat_ measurements at zero PPFD, [CO_2_] of 400 µmol mol^−^^1^ and mid-day growth temperatures.

### Leaf gas exchange responses to short-term temperature increases

On Days 10 and 11, when the growth chamber was heated along the temperature steps, the responses of leaf gas exchange to leaf temperature were simultaneously measured at PPFD of 1200 μmol m^−^^2^ s^−^^1^, [CO_2_] of 400 μmol mol^−^^1^ and a series of growth temperatures (26 °C, 30 °C, 34 °C, 38 °C and 42 °C). Four seedlings per water treatment (*n* = 4) were randomly chosen for the measurements.

### Estimation of *v*_cmax_

The *V*_cmax_ (µmol m^−^^2^ s^−^^1^) was estimated using the ‘one-point method’ which was described in De Kauwe *et al.* (2016).

(Eqn 1) $$V_{cmax}=\frac{A_{sat}}{\left(\frac{C_{i}-\Gamma^{*}}{C_{i}+K_{m}}-0.015\right)}$$

where *A*_sat_ is the leaf photosynthesis under saturating light (µmol m^−^^2^ s^−^^1^); *C*_i_ is the intercellular CO_2_ concentration (µmol mol^−^^1^); Γ* is the CO_2_ compensation point in the absence of mitochondrial respiration (µmol mol^−^^1^); *K*_m_ is the Michaelis–Menten constant, which is determined by

(Eqn 2) $$K_{m}=K_{c}\left(1+\frac{O_{i}}{K_{0}}\right)$$

where *K*_c_ is the Michaelis constant for CO_2_ (µmol mol^−^^1^); *K*_o_ is the Michaelis constant for O_2_ (mmol mol^−^^1^); *O*_i_ is the intercellular concentrations of O_2_. The determination of Γ*, *K*_c_, *K*_o_ and *O*_i_ can be estimated according to Bernacchi *et al.* (2001).

### Growth measurements

Height (*H*, cm) was measured from the stem base to the highest shoot tip and basal diameter (*D*, cm) was measured at 1-cm height. *D^2^H* (cm^3^) was calculated as *D^2^* times *H*, to estimate the aboveground growth (Kubiske *et al.* 2006).

### Leaf water potential measurements

Leaf water potential (*Ψ*_l_, MPa) measurements were taken on one leaf from each of the four seedlings per treatment (n  = 4) during the daytime throughout the experiment using a Scholander-type pressure chamber (PMS instruments, Corvallis, Oregon USA). The leaves were cut with a blade, wrapped in moist paper towel and measured for *Ψ*_l_ immediately after collection.

### Chlorophyll measurements

The non-destructive chlorophyll measurements were conducted using a portable chlorophyll meter (SPAD-502, Konica Minolta Optics Inc, Osaka, Japan). The SPAD values can reflect the relative chlorophyll content. Three leaves from each of six seedlings per treatments (n  = 6) were measured.

### Statistical analyses

Statistical analyses were performed with R 3.2.2 (R Core Team, 2015). The parameters including *A*_sat_, *g*_s_, *E*, *WUE*_i_, *V*_cmax_, *T*_air_, *T*_leaf_, *Ψ*_l_, SPAD value and growth during the course of the experiment were analysed using a linear mixed-effects model (package ‘nlme’ in R), with water treatment (well-watered vs. drought), temperature treatment (ambient temperature vs. heat wave), and days as categorical fixed effects. Seedling number was treated as a random effect in all analyses. As we showed the data of SWC only in drought treatment, we analysed SWC with temperature and days as fixed effects and seedling number as a random effect. At each time step, the main and interactive effects of water and temperature on parameters were analysed using two-way ANOVA and followed by one-way ANOVA when interactive effects were significant. During the period of recovery from drought (*i.e*. the recovery after 0 h, 1 h, 2 h, 8 h and 24 h), *A*_sat_, *g*_s_, *E*, and*Ψ*_l_ were analysed using a linear mixed-effects model, with temperature and days as fixed effects and seedling number as a random effect. Subsequently, Student’s *t-tests* were used to compare means between AD and HD treatments. The homoscedasticity and normality were checked prior to all the statistic analyses. In all cases, the results were considered significant if *P* ≤ 0.05.

## Results

### Temperature and soil water conditions

The air temperature was elevated by about 16 °C during the 7-d heat wave period (*i.e*. from Day 10 to Day 17) and returned to ambient temperature level afterwards. The leaf temperature varied in concert with air temperature throughout the experiment (Fig. 1B), but the leaf temperature in HW treatment was elevated to a lesser degree during the heat wave period than that in HD treatment. After the heat wave, leaf temperature was unable to go back to the target temperature, about 1 °C higher than the ambient temperature treatments. The SWC declined from 60 % at the beginning to about 20 % on Day 6 and was maintained at this level until seedlings were rehydrated on Day 20 (Fig. 1C). During the heat wave period, the SWC in HD treatment was not lower than that in AD treatment, indicating that soil water conditions were similar between temperature treatments.

## Growth

**Figure 2:**
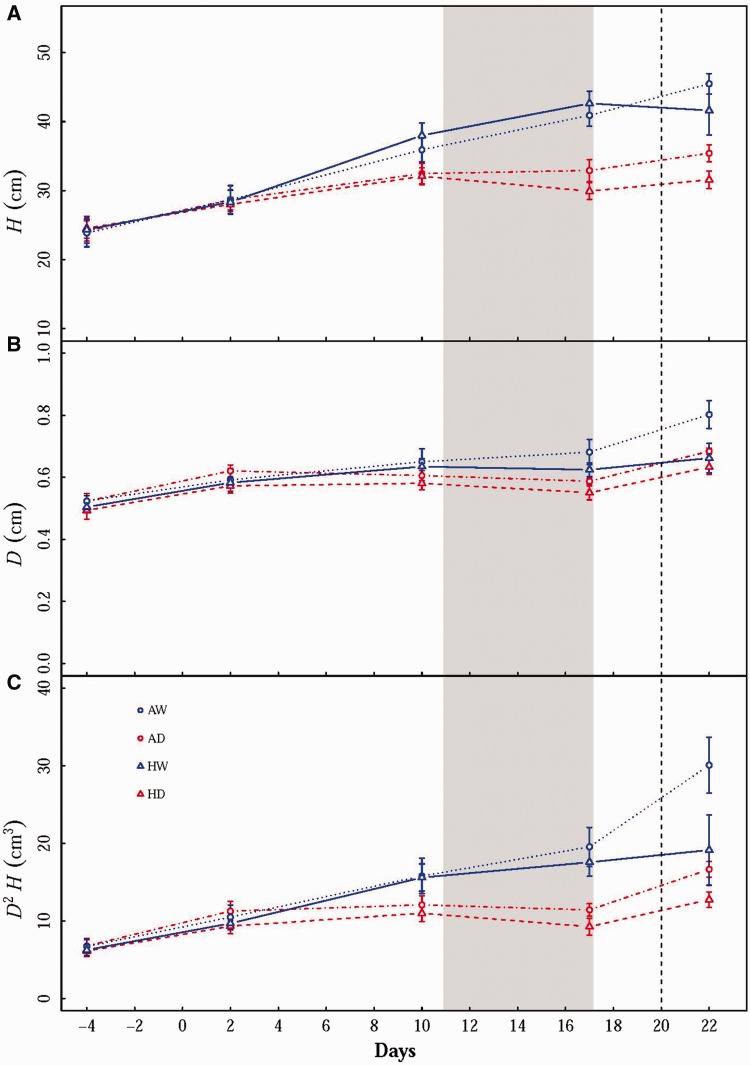
(A) Height (*H*), (B) basal diameter (*D*) and (C) estimated stem growth (*D^2^H*) of *Solanum lycopersicum* seedlings throughout the experiment. Values are means ± SE (*n* = 6). The grey area represents the period during which a 7-day 42 °C heat wave was applied. The vertical line represents the day when droughted seedlings were rehydrated. Treatments: AW–Ambient temperature plus well watered; AD– Ambient temperature plus drought; HW–Heat wave plus well watered; HD–Heat wave plus drought.

The growth parameters of *S. lycopersicum* seedlings were substantially reduced by drought stress during the experiment (Fig. 2, Table 1). At the end of the heat wave period (*i.e*. Day 17), growth parameters were not changed by the heat wave treatments. Hence, drought had a primary impact on seedling growth. At the end of the experiment (*i.e*. Day 22), heat wave had remarkably reduced seedling *D^2^H* at both water conditions (Two-way ANOVA: *P * =* *0.017) (Fig. 2C).

**Table 1.** Summary of linear mixed model analysis of effects of water, temperature treatment and time on measured parameters of *solanum lycopersicum* seedlings over the entire experimental period. Significant values are shown in bold (*P* < 0.05).

Parameter		Water	Temp	Days	Water:Temp	Water:Days	Temp: Days	Water: Temp:Days
*T*_air_	numDF	1	1	8	1	8	8	8
	denDF	12	12	96	12	96	96	96
	*P*	0.2938	**<0.0001**	**<0.0001**	**0.0196**	**0.0020**	**<0.0001**	**0.0213**
*T*_leaf_	numDF	1	1	8	1	8	8	8
	denDF	12	12	96	12	96	96	96
	*P*	**0.0059**	**<0.0001**	**<0.0001**	0.1424	**<0.0001**	**<0.0001**	**0.0008**
*H*	numDF	1	1	4	1	4	4	4
	denDF	20	20	80	20	80	80	80
	*P*	0.3966	0.8369	**<0.0001**	0.8145	**<0.0001**	**0.0001**	**0.0085**
*D*	numDF	1	1	4	1	4	4	4
	denDF	20	20	80	20	80	80	80
	*P*	0.0874	0.0769	**<0.0001**	0.8437	**<0.0001**	**0.0001**	**0.0026**
*D*^2^*H*	numDF	1	1	4	1	4	4	4
	denDF	20	20	80	20	80	80	80
	*P*	**0.0001**	0.3519	**<0.0001**	0.4035	**<0.0001**	**0.0001**	**0.0073**
*A*_sat_	numDF	1	1	8	1	8	8	8
	denDF	12	12	95	12	95	95	95
	*P*	**<0.0001**	**0.0001**	**<0.0001**	**0.0057**	**<0.0001**	**<0.0001**	**0.0014**
*g*_s_	numDF	1	1	8	1	8	8	8
	denDF	12	12	95	12	95	95	95
	*P*	**<0.0001**	**<0.0001**	**<0.0001**	**0.0001**	**<0.0001**	**<0.0001**	**<0.0001**
*E*	numDF	1	1	8	1	8	8	8
	denDF	12	12	95	12	95	95	95
	*P*	**<0.0001**	**<0.0001**	**<0.0001**	**0.0003**	**<0.0001**	**<0.0001**	**<0.0001**
*WUE*_i_	numDF	1	1	8	1	8	8	8
	denDF	12	12	96	12	96	96	96
	*P*	**0.0001**	**<0.0001**	**<0.0001**	**0.0001**	**<0.0001**	**<0.0001**	**<0.0001**
*V*_cmax_	numDF	1	1	9	1	9	9	9
	denDF	13	13	105	13	105	105	105
	*P*	**<0.0001**	0.1608	**<0.0001**	0.9778	**<0.0001**	**<0.0001**	**<0.0001**
SPAD	numDF	1	1	5	1	5	5	5
	denDF	20	20	100	20	100	100	100
	*P*	0.1949	**0.0475**	**<0.0001**	0.0722	**0.0242**	**<0.0001**	**0.0217**
*Ψ*_l_	numDF	1	1	7	1	7	7	7
	denDF	13	13	83	13	83	83	83
	*P*	**<0.0001**	0.0926	**<0.0001**	0.3584	**<0.0001**	0.2181	0.7756

Numerator and denominator df are the numerator and denominator degrees of freedom for the *F*-tests.

### Time course of leaf physiological responses

**Figure 3:**
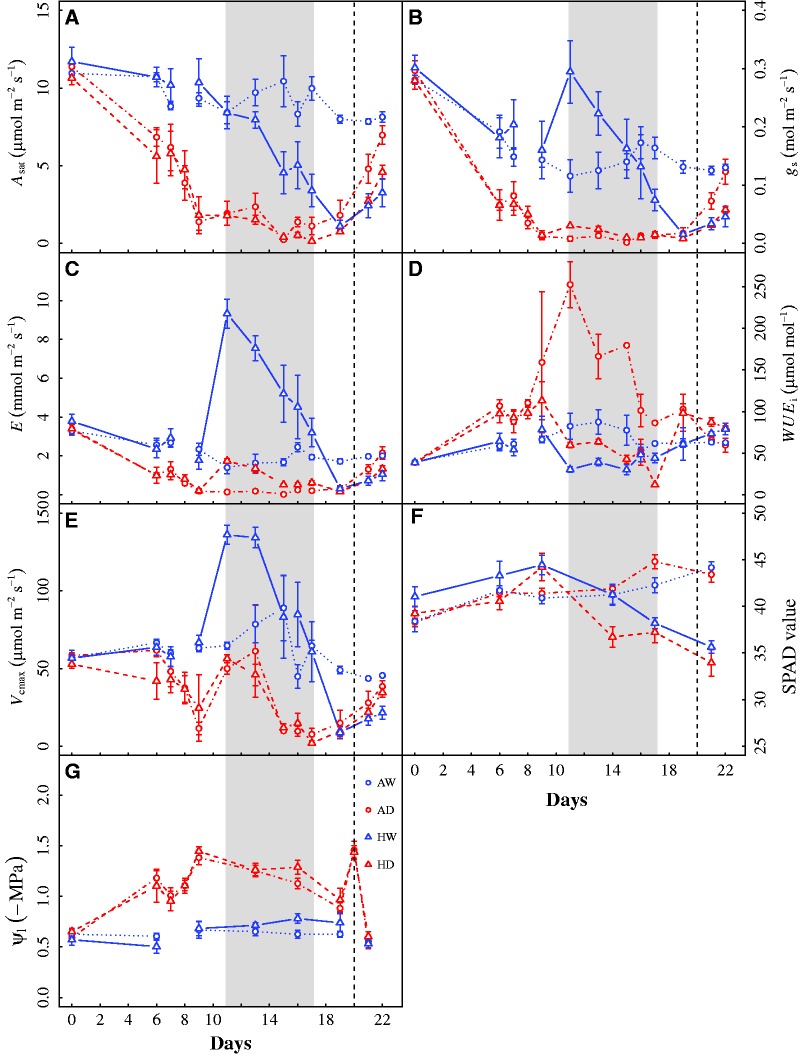
(A) Leaf photosynthesis under saturating light (*A*_sat_), (B) stomatal conductance (*g*_s_), (C) transpiration (*E*), (D) instantaneous water use efficiency (*WUE*i), (E) the estimated maximum carboxylation capacity (*V*_cmax_), (F) SPAD value and (G) leaf water potential (*Ψ*_l_) of *Solanum lycopersicum* seedlings throughout the experiment. Values are means ± SE (*n* = 4–6). The grey area represents the period during which a 7-day 42 °C heat wave was applied. The vertical line represents the day when droughted seedlings were rehydrated. Treatments: AW–Ambient temperature plus well watered; AD– Ambient temperature plus drought; HW–Heat wave plus well watered; HD–Heat wave plus drought.

*A*_sat_ of *S. lycopersicum* seedlings in AW control did not change too much over time, while *A*_sat_ in HW treatment declined substantially as heat wave progressed, recovering to only 39 % of AW control in the end (Fig. 3A, Table 1). These results reflect that the heat wave had significant negative impacts on photosynthesis. *A*_sat_ was reduced in the drought treatments compared with the well watered treatments at both temperatures, maintaining lower than 10 % of pre-drought values until the seedlings were rehydrated. During the pre-heat wave and heat wave period (*i.e*. from Day 0 to Day 17), *A*_sat_ in droughted seedlings did not differ between temperature treatments, indicating that photosynthesis in response to drought was not altered by heat wave. By contrast, the recovery of *A*_sat_ from drought was slower in HD treatment than AD treatment (*t*-test: *P *<* *0.05). For instance, after two days rehydration, *A*_sat_ in AD treatment almost returned to the value of the control, while that in HD treatment was only 71 % of *A*_sat_ in AD treatment.

For the entire experimental period, *g*_s_ appeared to be one of the dominant factors in determining *A*_sat_ responses (Fig. 3B, [see **Supporting Information—Fig. S1**]). During the 7-day heat wave, *g*_s_ in HW treatment was initially increased by 81 % compared with the pre-heat wave value, but declined sharply as heat wave prolonged. At the end of the experiment, full recovery of *g*_s_ in HW treatment was not observed. *g*_s_ were similar between AD and HD treatments prior to rehydration, but *g*_s_ in AD treatment recovered faster from drought than that in HD treatment (t-test: *P *<* *0.05). The time course responses of *E* were similar to those of *g*_s_ in all treatments (Fig. 3C, Table 1).

*WUE*_i_ was significantly enhanced in the drought treatments compared with the well watered treatments at both temperatures over the entire experimental period (Fig. 3D, Table 1). During the 7-day heat wave, *WUE*_i_ in HW and HD treatments were both substantially reduced compared with the ambient temperature treatments, suggesting that the heat wave had large negative impacts on *WUE*_i_.

*V*_cmax_ in AD and HD treatments declined sharply compared with the well watered treatments when the target drought stress was achieved (Fig. 3E, Table 1). *V*_cmax_ of droughted plants was maintained at relatively low values until plants were rehydrated, afterwards exhibiting substantial recovery. Over the entire experimental period, *V*_cmax_ did not significantly differ between AD and HD treatments, suggesting that the heat wave had minimal effects on *V*_cmax_ when the stomata were almost closed. However, *V*_cmax_ in HW treatment showed large variations (Fig. 3E). It was increased to nearly 200% of *V*_cmax_ in AW control at the onset of the heat wave (*i.e*. Day 11), while it had continuous declines as the heat wave was prolonged. Although *V*_cmax_ in HW treatment was recovered to some extent after the release of heat wave, it could not return to the control value even at the end of the experiment.

SPAD value (*i.e*. relative chlorophyll content) did not change too much in all treatments prior to the heat wave, while it had a pronounced reduction in the heat wave treatments (*i.e*. HW and HD) compared with the ambient temperature treatments (*i.e*. AW and AD), maintaining only 80% of ambient temperature values in the end (Fig. 3F, Table 1).

*Ψ*_l_ was generally more negative in the drought treatments (*i.e*. AD and HD treatments; −1.3 MPa on average) than the well watered treatments (*i.e*. AW and HW treatments; -0.65 MPa on average) (Fig. 3G, Table 1), reflecting the moderate drought stress on seedlings. Yet, heat wave did not lead to overall reduction in *Ψ*_l_ than did the ambient temperature over the course of the entire experiment, except for Day 16 (Two-way ANOVA: *P * =* *0.011).

### Leaf gas exchange as function of leaf temperature

**Figure 4:**
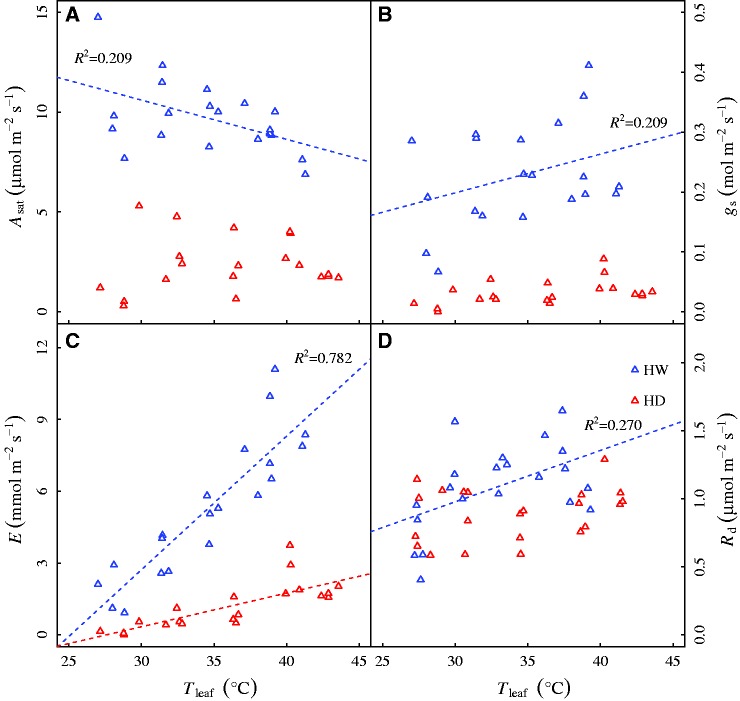
(A) Leaf photosynthesis under saturating light (*A*_sat_), (B) stomatal conductance (*g*_s_), (C) transpiration (*E*) and (D) dark respiration (*R*_d_) of *Solanum lycopersicum* seedlings grown under well-watered and drought conditions as function of leaf temperature during the period of short-term temperature increases. Data points are raw data of the measured variables at growth temperature of 26 °C, 30 °C, 34 °C, 38 °C and 42 °C. Data are fitted with linear functions (y = ax + b) and only significant relationships are showed. The correlation coefficients (*R*^2^) are also given. Treatments: HW–Heat wave plus well watered; HD–Heat wave plus drought.

*A*_sat_, *g*_s_ and *R*_d_ of *S. lycopersicum* seedlings had weak but significant linear relationships with leaf temperature, only in HW treatment (Fig. 4). By contrast, *E* of both well watered and drought seedlings had pronounced positive relationships with leaf temperature (Fig. 4C), indicating that short-term increases in temperature had greater impacts on *E* than other gas exchange traits.

### Physiological recovery from drought

**Figure 5:**
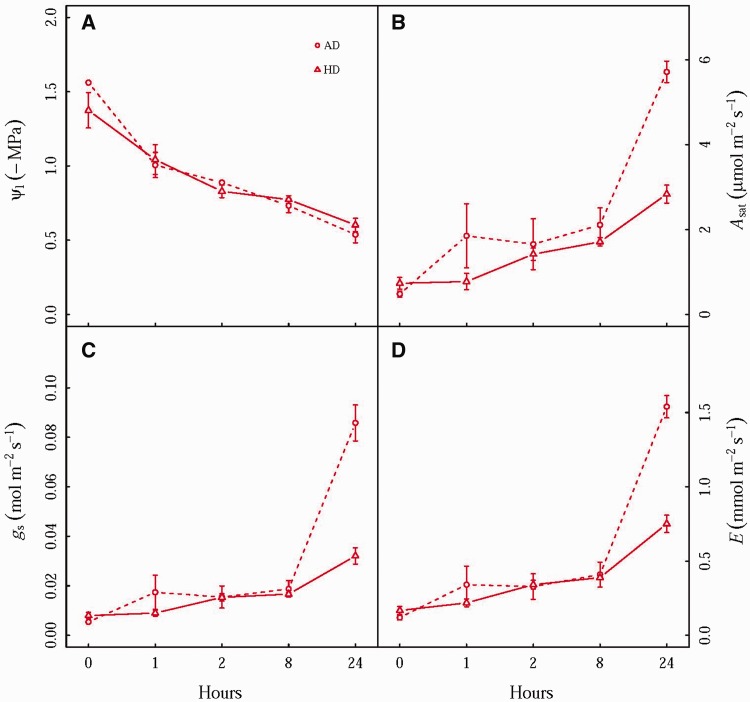
The recovery following drought of (A) leaf water potential (*Ψ*_l_), (B) leaf photosynthesis under saturating light (*A*_sat_), (C) stomatal conductance (*g*_s_) and (D) transpiration (*E*) of *Solanum lycopersicum* seedlings grown under ambient temperature and 7-d 42 °C heat wave treatments. Values are means ± SE (*n* = 4). Hours represent 0, 1, 2, 8, 24 h after the recovery from drought. Treatments: AD–Ambient temperature plus drought; HD–Heat wave plus drought.

**Figure 6:**
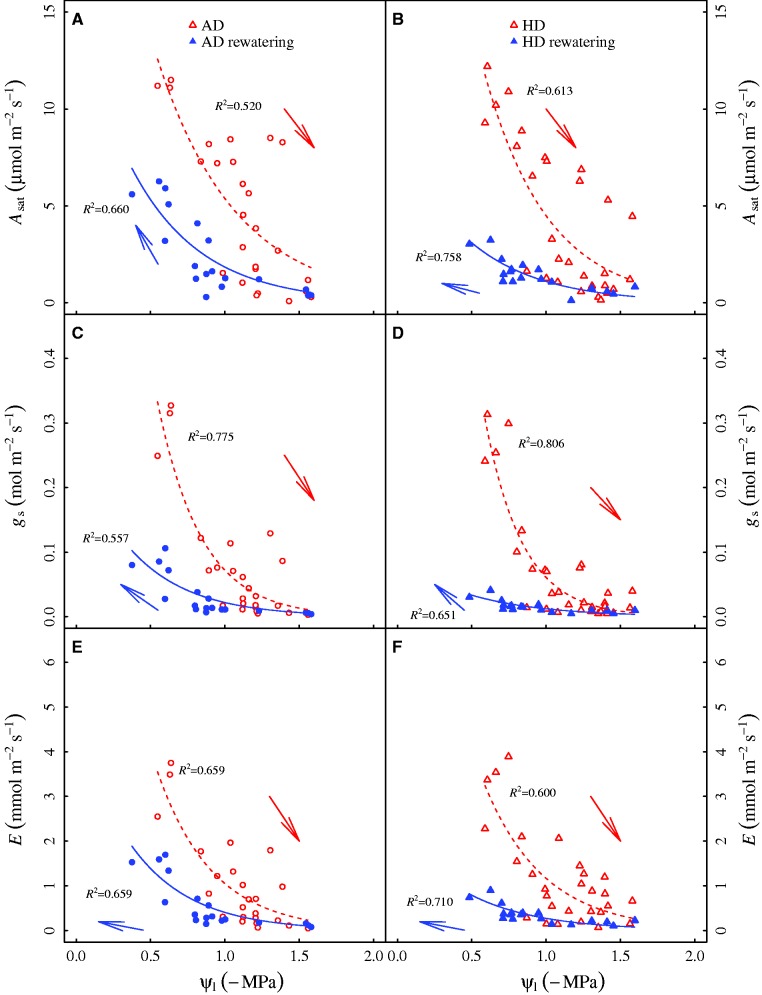
Leaf photosynthesis under saturating light (*A*_sat_), stomatal conductance (*g*_s_) and transpiration (*E*) of *Solanum lycopersicum* seedlings grown under ambient temperature (A, C and E) and 7-d 42 °C heat wave treatments (B, D and F) as function of leaf water potential (*Ψ*_l_) during the periods of drought stress and the following recovery. Data points are raw data of the measured variables. Data are fitted with exponential functions: y = a × e ^(-bx)^ (*P *<* *0.01 for all parameters). The correlation coefficients (*R*^2^) are also given. Red and open symbols represent values during the drought stress, while blue and closed symbols represent values during the recovery from drought. Treatments: AD–Ambient temperature plus drought; HD–Heat wave plus drought.

The leaf water potential had a fast recovery from drought, returning to well watered control values in about 8 h after rehydration, similarly in AD and HD treatments (Fig. 5A, Table 2). Nevertheless, leaf gas exchange was not fully recovered even after 24 h rehydration (Fig. 5B–D, Table 2), reflecting the hysteresis in the response of leaf gas exchange to leaf water potential. More interestingly, after 24 h recovery, *A*_sat_, *g*_s_ and *E* in AD was 210 %, 260 % and 214 % higher than those in HD treatment, respectively, suggesting that heat wave delayed the recovery of leaf gas exchange. These results are explained in Fig. 6, which shows leaf gas exchange as a function of leaf water potential during drought stress and the 24-h recovery from drought. As leaf water potential declined (*i.e*. to more negative values), gas exchange exhibited similar reductions between AD and HD treatment. After rehydration, however, seedlings in AD treatment exhibited sharper recovery of leaf gas exchange, particularly when leaf water potential was higher than -1 MPa. Generally, these results suggest that despite the fast recovery of leaf water potential, the recovery of leaf gas exchange lagged, to an even greater degree in HD treatment than AD treatment, suggesting the coordination of leaf gas exchange and leaf water potential was shifted between AD and HD treatments.

**Table 2.** Summary of linear mixed model analysis of effects of temperature treatment and recovery time on measured parameters of *solanum lycopersicum* seedlings during the 24-h recovery from drought. Here, only values in AD and HD treatment were analysed to examine whether initial temperature treatment modifies the recovery of physiological parameters of seedlings from drought. Hours represent 0, 1, 2, 8 and 24 h after rehydration. Significant values are shown in bold (*P* < 0.05).

Parameter		Temp	Hours	Temp:Hours
*Ψ*_l_	numDF	1	4	4
	denDF	7	24	24
	*P*	0.7914	**<0.0001**	0.2376
*A*_sat_	numDF	1	4	4
	denDF	7	24	24
	*P*	0.6936	**<0.0001**	**<0.0001**
*g*_s_	numDF	1	4	4
	denDF	7	24	24
	*P*	0.5246	**<0.0001**	**<0.0001**
*E*	numDF	1	4	4
	denDF	7	24	24
	*P*	0.5429	**<0.0001**	**<0.0001**

Numerator and denominator df are the numerator and denominator degrees of freedom for the *F*-tests.

## Discussion

This study investigated the individual and interactive effects of drought and heat wave on leaf physiology and growth of *S. lycopersicum* seedlings, and how leaf physiology recovered from the combined stress. Our results showed that the 7-day heat wave treatment (*i.e*. HW), as predicted in the first hypothesis, reduced growth, leaf gas exchange rates, *V*_cmax_ and water use efficiency compared with ambient temperature control (*i.e*. AW), while it did not have significant impacts on leaf water potentials. Compared with well watered conditions, drought stress led to stomatal closure, lower *V*_cmax_, reduced growth and more negative leaf water potentials, similarly in AD and HD treatments. However, the observed lower relative chlorophyll content in HD treatment can partially support our second hypothesis that heat wave would exacerbate drought stress. Our results also demonstrated that recovery of leaf gas exchange lagged behind water potential recovery and leaf gas exchange exhibited much slower post-drought recovery in HD treatment than AD treatment, which agrees with our third hypothesis.

### Plant physiological responses during and post single heat wave treatment

The 7-day + 16 °C single factor heat wave (*i.e*. 42 °C) had negative impacts on the growth of *S. lycopersicum* seedlings, which is consistent with previous studies (*e.g*. Bauweraerts *et al.* 2014; Ruehr *et al.* 2016). Under high soil water availability, despite the initial sharp rise in leaf stomatal conductance and transpiration at the onset of the heat wave, photosynthesis declined gradually in parallel with stomatal conductance as heat wave progressed, maintaining a relatively low leaf level water use efficiency. Even 2 days after the relief of a heat wave, photosynthesis and stomatal conductance exhibited continued declines. These results indicate that the effect of heat wave on plant performance depends not only on the duration of heat exposure but also on the heat stress legacy (Teskey *et al.* 2014; Mitchell *et al.* 2016). Apparently, the reduction in photosynthesis in the present study was partly attributed to stomatal closure. It is noted that leaf water potentials did not significantly differ between ambient temperature and heat wave treatments or among pre-, during and post heat wave period. This is not surprising because we well watered the plants in HW treatment daily to maintain the high soil water availability. The observed stomatal closure associated with heat wave is thereby not likely to be induced by soil water deficit. However, the close negative correlation of stomatal conductance with leaf-to-air VPD (Linear regression; gs  =-0.15 × VPD + 0.77; *R*^2^= 0.76) illustrated that the high VPD associated with high temperature could have inhibited stomatal conductance. It has been demonstrated that the gradient in water potential between guard cells and epidermal cells rather than bulk leaf water potential, can affect stomatal responses to high VPD (Bunce, 1997). Abscisic acid (ABA) may also be involved in this process (McAdam & Brodribb, 2015).

In addition to stomatal limitation, the non-stomatal limitation could also play the role in determining photosynthesis in response to the heat wave. During the 7-day heat wave period, leaf senescence and reduction in chlorophyll content were observed in well watered plants, which is supported by other studies (Marchand *et al.* 2006; Wang *et al.* 2015). For example, Wang *et al.* (2015) found that tomato grown under 42 °C for 24 h had significantly lower chlorophyll content than that grown under 25 °C. In the present study, the very high leaf temperature (*i.e*. about 40 °C) could have caused leaf damage, thereby limiting photosynthesis. There is also evidence that *V*_cmax_ was substantially reduced in HW treatment compared with AW control towards the end of the heat stress, indicating that effects of non-stomatal limitation on photosynthesis were progressively enhanced. Together, it is suggested that the non-stomatal limitation can be a co-limiting factor affecting photosynthesis responses under heat stress and its contribution may vary depending on the duration of exposure to stress. The partial recovery of photosynthesis, stomatal conductance and *V*_cmax_ in the end indicated that the 42 °C heat stress had remarkably negative impacts on *S. lycopersicum* seedlings even after the heat stress was relieved. It is worth for further studies to quantify the relative contribution of stomatal and non-stomatal limitation on photosynthesis under stress and the following recovery, for better modeling plant responses to future climate change (Zhou *et al.* 2013).

### Plant physiological responses in combination of drought and heat wave

Drought stress in the present study appeared to have substantially negative impacts on plant growth, leaf gas exchange, *V*_cmax_ and leaf water relations, generating a greater degree of reduction in the above traits than well watered treatments, under either ambient temperature or heat wave treatment. In agreement with other studies (Zhou *et al.* 2013, 2014), the results also showed that the reduction in photosynthesis due to water stress was influenced by a combination of stomatal and non-stomatal limitations. Alongside current evidence (Carmo-Silva *et al.* 2012; Bauweraerts *et al.* 2013, 2014; Duan *et al.* 2013; Ruehr *et al.* 2016), our results confirmed that water availability has a dominant role in determining plant physiological responses. Drought and heat stress are usually linked and heat stress has been found to exacerbate the negative impacts of drought on plant physiology (Duan *et al.* 2014; Teskey *et al.* 2014; Adams *et al.* 2015). The high VPD associated with heat stress often accelerates evapotranspiration, thereby aggravating soil water depletion. In the present study, to minimize the potential confounding effects of heat wave on soil water conditions, we maintained soil water content through the drought period, which is evident in the similar values of soil water content and leaf water potentials between the two temperature treatments. Since leaf stomata were almost completely closed, the difference of leaf gas exchange was not observed between AD and HD treatment. However, the reduced chlorophyll content in HD treatment compared with AD treatment reflected the fact that the combined stress of drought and heat wave resulted in greater leaf damage than drought alone (De Boeck *et al.* 2011). Therefore, the above results demonstrated that the combined negative effects of drought and heat wave on plant physiological responses were much larger than single drought stress effect.

### Post-drought recovery under ambient temperature and heat wave

The present study has added into the current uncertain knowledge of how plant physiology recovers from the combined stress of drought and heat wave. More importantly, the present study examined a much less studied aspect of how leaf gas exchange and water relations are coordinated during the recovery from weather extremes. After rehydration, leaf water potential recovered at a higher rate than leaf gas exchange traits in both AD and HD treatments, indicating that the recovery of leaf gas exchange was decoupled with that of leaf water potential (see Brodribb and McAdam 2013; Martorell *et al.* 2014). Thus, the slower recovery of leaf photosynthesis and stomatal conductance was likely to be explained by non-hydraulic factors other than leaf water potential. ABA has been suggested as a contributor in regulating stoma re-opening during recovery from drought (Brodribb and McAdam 2013). Future determination of ABA will be helpful to understand plant stomatal behaviour during drought stress and the following recovery.

More interestingly, recovery of leaf gas exchange was much slower in HD treatment than AD treatment, which suggests that previous imposed heat wave delayed the post-drought recovery of leaf gas exchange. This finding is in line with the recent study on black locust tree seedlings (Ruehr *et al.* 2016), reflecting that the recovery of leaf gas exchange largely depends on the degree of previous drought stress. Particularly, the slower recovery of photosynthesis in HD treatment than in AD treatment can be attributed to stomatal and non-stomatal limitations (*i.e*. reduced chlorophyll content). Further studies are required to determine the detailed physiological processes and contributors during the recovery of plant from weather extremes. Altogether, our study demonstrated that heat wave largely affected the recovery of leaf gas exchange from drought in *S. lycopersicum* seedlings and highlighted the importance of studies on the interactive effects of drought and heat wave on plant recovery.

## Conclusions

This study provides new insights into how tomato seedlings recover from the combined stress of drought and heat wave. Heat wave and drought both had significant negative impacts on photosynthetic responses of *S. lycopersicum* seedlings through stomatal and non-stomatal limitations. Heat wave in combination with drought had greater negative effects on plants than single drought stress. Leaf gas exchange of seedlings grown in the combination of drought and heat wave exhibited slower post-stress recovery and its recovery was decoupled with water potential recovery. Therefore, our study demonstrated that drought and heat wave in combination had significant negative effects on plant growth and leaf physiology during and post stress. Stomatal and non-stomatal limitations to photosynthesis need to be considered for more accurately predicting crop responses to environmental stresses. Results from our growth chamber study may not be easily extrapolated to field studies. However, our study confirms that drought and heat wave are interactively linked and more field studies are needed to uncover the particular physiological mechanisms of how crops respond to cyclic stress of drought and heat wave, which will provide more reliable predictions of crop responses under future climates characterized by more frequent weather extremes.

## Sources of Funding 

This work was supported by grants from the National Natural Science Foundation of China (31600483; 31570444; 31360175) and Jiangxi Provincial Department of Education (KJLD12097; GJJ14744; GJJ151097) and Gan-Po 555 Talent Project.

## Contributions by the Authors

H.D., J.W. and H.F. conceived the experiment, H.D., X.Y. and Z.X. conducted the experiment, H.D., J.W., G.H, S.Z., W.L. and Y.L. analysed the results, H.D. wrote the manuscript with input from all of the other authors.

## Conflicts of Interest Statement

None declared.

## Supporting Information

The following additional information is available in the online version of this article —

**Figure S1.** Leaf photosynthesis under saturating light (*A*_sat_) as a function of stomatal conductance (*g*_s_) of *Solanum lycopersicum* seedlings throughout the experiment. Data points are raw data of the measured variables. Data are fitted with exponential saturation functions: y  = a × (1−e^(-bx)^). The correlation coefficient (*R*^2^) in each treatment is 0.402 (AW), 0.744 (HW), 0.936 (AD) and 0.926 (HD), respectively. The fitted functions are not significant differences among treatments. Treatments: AW–Ambient temperature plus well watered; AD–Ambient temperature plus drought; HW–Heat wave plus well watered; HD–Heat wave plus drought.

**Figure S2.** (A) Leaf-to-air vapour deficit (VpdL) and (B) ratio of intercellular to atmospheric [CO_2_] (*C*_i_/*C*_a_) of *Solanum lycopersicum* seedlings throughout the experiment. Values are means ± SE (*n* = 4–6). The grey area represents the period during which a 7-day 42 °C heat wave was applied. The vertical line represents the day when droughted seedlings were rehydrated. Treatments: AW–Ambient temperature plus well watered; AD– Ambient temperature plus drought; HW–Heat wave plus well watered; HD–Heat wave plus drought.

**Table S1.** The raw data of leaf gas exchange and estimated *V*_cmax_ in this paper.

## Supplementary Material

Supplementary Data
