# Supplementary material for: Individual and interactive effects of drought and heat on leaf physiology of seedlings in an economically important crop
Source: AoB Plants. 2016 Dec 23;9(1):plw090. doi: 10.1093/aobpla/plw090 (PMC5391698; doi:10.1093/aobpla/plw090)

**Figure S1** Leaf photosynthesis under saturating light (*A*_sat_) as function of stomatal conductance (*g*_s_) of *Solanum lycopersicum* seedlings throughout the experiment. Data points are raw data of the measured variables. Data are fitted with exponential saturation functions: y=a×(1-e^(-bx)^). The correlation coefficient (*R*^2^) in each treatment is 0.402 (AW), 0.744 (HW), 0.936 (AD) and 0.926 (HD), respectively. The fitted functions are not significant different among treatments. Treatments: AW--Ambient temperature plus well watered; AD-- Ambient temperature plus drought; HW--Heat wave plus well watered; HD--Heat wave plus drought.


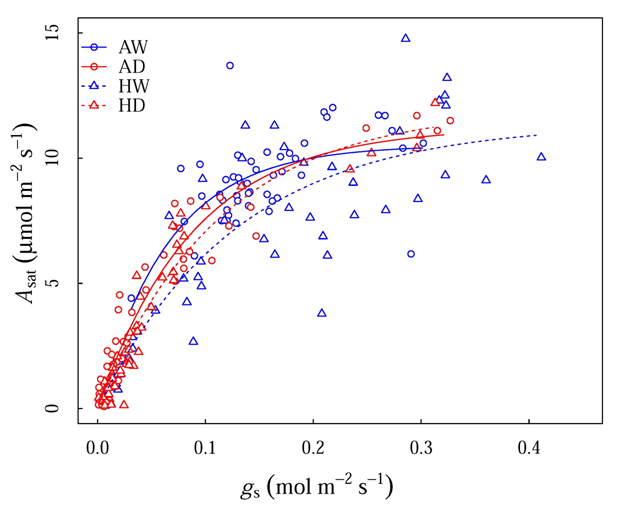


**Figure S2** (a) Leaf-to-air vapour deficit (VpdL) and (b) ratio of intercellular to atmospheric [CO_2_] (*C*_i_/*C*_a_) of *Solanum lycopersicum* seedlings throughout the experiment. Values are means ± SE (n = 4-6). The grey area represents the period during which a 7-day 42 °C heat wave was applied. The vertical line represents the day when droughted seedlings were rehydrated. Treatments: AW--Ambient temperature plus well watered; AD-- Ambient temperature plus drought; HW--Heat wave plus well watered; HD--Heat wave plus drought.


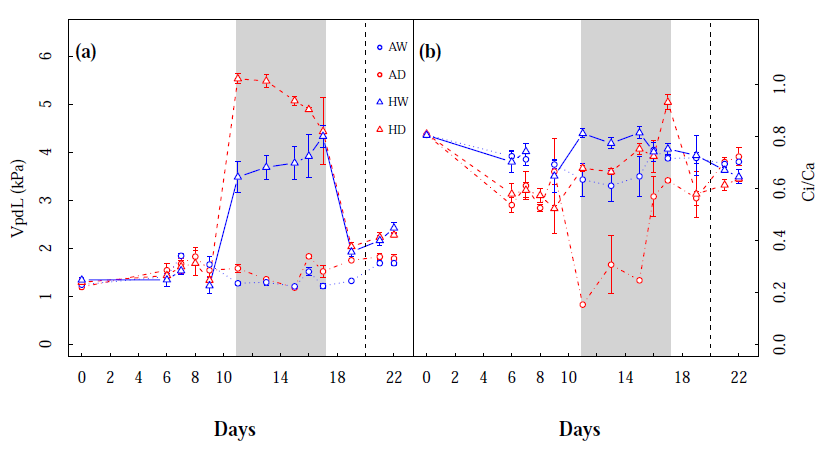

Supplement: Supplementary Data [file plw090_Supp.zip › Supporting information Figures S1 and S2.docx]
